# Thoracoscopic esophagectomy with subcarinal lymph node dissection in the prone position for esophageal cancer with a right top pulmonary vein

**DOI:** 10.1093/jscr/rjad462

**Published:** 2023-08-14

**Authors:** Shinya Mikami, Takeharu Enomoto, Jun Shimada, Masaki Hiwatari, Yoshitsugu Tsukamoto, Yasuhito Hisatsune, Sae Kimura, Hirotaka Arifuku, Saori Umezawa, Takehito Otsubo

**Affiliations:** Department of Gastrointestinal and General Surgery, St. Marianna University School of Medicine, Kawasaki, Kanagawa 216-8511, Japan; Department of Gastrointestinal and General Surgery, St. Marianna University School of Medicine, Kawasaki, Kanagawa 216-8511, Japan; Department of Gastrointestinal and General Surgery, St. Marianna University School of Medicine, Kawasaki, Kanagawa 216-8511, Japan; Department of Gastrointestinal and General Surgery, St. Marianna University School of Medicine, Kawasaki, Kanagawa 216-8511, Japan; Department of Gastrointestinal and General Surgery, St. Marianna University School of Medicine, Kawasaki, Kanagawa 216-8511, Japan; Department of Gastrointestinal and General Surgery, St. Marianna University School of Medicine, Kawasaki, Kanagawa 216-8511, Japan; Department of Gastrointestinal and General Surgery, St. Marianna University School of Medicine, Kawasaki, Kanagawa 216-8511, Japan; Department of Gastrointestinal and General Surgery, St. Marianna University School of Medicine, Kawasaki, Kanagawa 216-8511, Japan; Department of Gastrointestinal and General Surgery, St. Marianna University School of Medicine, Kawasaki, Kanagawa 216-8511, Japan; Department of Gastrointestinal and General Surgery, St. Marianna University School of Medicine, Kawasaki, Kanagawa 216-8511, Japan

**Keywords:** right top pulmonary vein, esophageal cancer, thoracoscopic esophagectomy, prone position

## Abstract

The right top pulmonary vein (RTPV), a rare pulmonary vein (PV) variant draining the right upper lobe, arises independently from the right superior PV, travels posterior to the right bronchial tree and drains directly into the left atrium (LA) or another PV. We report an RTPV discovered on preoperative computed tomography (CT) scanning in a 60-y-old man who subsequently underwent prone thoracoscopic esophagectomy and subcarinal lymph node dissection. The preoperative CT scan showed an anomalous vessel 7.8 mm in diameter arising from the right upper lobe, running posterior to the right main bronchus (RMB), and draining directly into the LA. To our best knowledge, this is the largest reported RTPV (7.8 mm in diameter) and is an extremely rare variant, passing posterior to the RMB and draining into the LA.

## INTRODUCTION

The right top pulmonary vein (RTPV), a rare anomalous vessel draining the right upper lobe region, arises independently from the right superior pulmonary vein (PV), runs posterior to the right main bronchus (RMB) or right intermediate bronchus (RIB), and drains into the left atrium (LA) or another PV [1]. This anomalous route takes the vessel posterior to the RMB or RIB, and caution is required to avoid vascular injury during subcarinal lymph node dissection (LND) as part of the surgical treatment of esophageal cancer. We report a case in which preoperative computed tomography (CT) led to the diagnosis of an RTPV in a patient who subsequently underwent prone thoracoscopic esophagectomy with subcarinal LND.

## CASE REPORT

**Figure 1:**
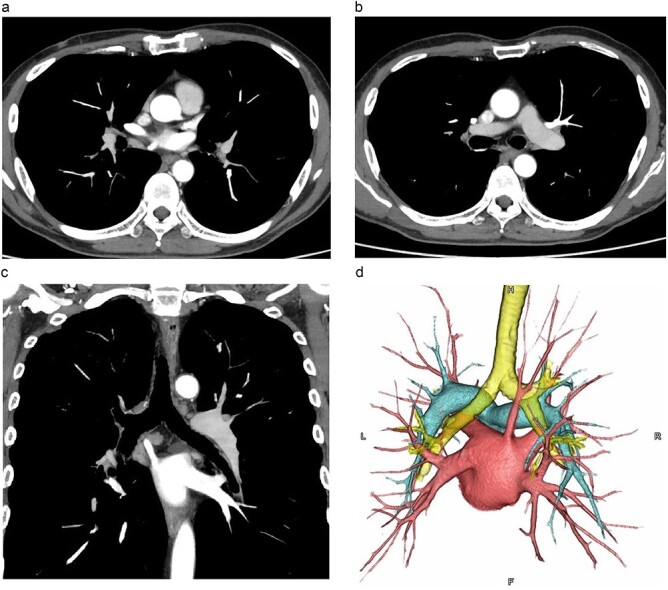
CT images. (**a**, **b**: axial images, **c**: coronal image, **d**: 3D image). The vessel was observed arising from the PVs draining the posterior upper lobe, running posterior to the RMB, and draining into the LA.

The patient was a 60-y-old man diagnosed as having squamous cell carcinoma measuring approximately 40 mm in the middle thoracic esophagus (Mt). The CT scan showed an anomalous vessel 7.8 mm in diameter arising from the right upper lobe, running posterior to the right bronchial tree, and emptying directly into the LA (Fig. 1a–c), which was correctly identified as a RTPV.

We then diagnosed the patient as having cancer of the Mt, staged as cT1b N0M0 cStage IA (UICC, 8th ed.) accompanied by an RTPV.

**Figure 2:**
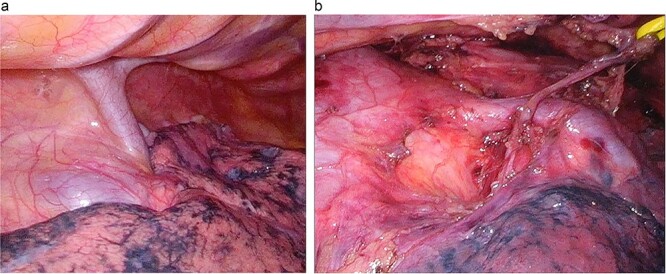
Surgical findings. (**a**) A large-caliber, anomalous PV was running posterior to the RMB. (**b**) After LND: the anomalous vessel is draining into the LA.

We placed the patient in the prone position and induced general anesthesia. Five thoracoscopic ports were placed under artificial CO_2_ pneumothorax (CO_2_ pressure 8 mmHg). When examining the thoracic cavity, we saw a large-caliber inflowing PV running dorsal to the RMB and projecting beyond the mediastinal pleura (Fig. 2).

During surgery, we mobilised the esophagus in the middle and lower mediastinum and started lower mediastinal LND. Normally, dissection is performed by advancing dissection in a cranial direction along the ventral esophagus and parietal pericardium along a plane continuous with that observed in the lower mediastinum. It was difficult to perform bilateral subcarinal LND along the same plane due to the presence of the anomalous RTPV. Therefore, we first performed fractional dissection of the right subcarinal LN. We then mobilised and dissected the left subcarinal LN, preserving the RTPV. We could then perform upper mediastinal LND and conclude the thoracic procedure. The patient was then transferred to the supine position, and abdominal LND and formation of the gastric tube were performed laparoscopically. The gastric tube was elevated via a retrosternal route, and we created an esophagogastric anastomosis. Surgery lasted 394 min, and blood loss was 45 ml. There were no postoperative complications, the clinical course was unremarkable, and the patient was discharged on postoperative day 17.

## DISCUSSION

The RTPV and other pulmonary venous anomalies have generated discussion among cardiothoracic surgeons and cardiologists, particularly with regard to pulmonary venous catheter ablation to treat atrial fibrillation [2]. In 2004, Lickfett et al. evaluated patients undergoing catheter ablation to treat atrial fibrillation using three-dimensional (3D) magnetic resonance angiography. They defined the RTPV as an anatomically anomalous PV emptying directly into the LA and reported its presence in 3.2% (3/91) of patients [1].

In 2013, Akiba et al. analyzed 303 3D CT images, finding variant right PVs in 10 subjects (3.3%), with eight of them (2.6%) presenting with a right upper lobe vein posterior to the RIB, one (0.33%) with a single vein posterior to the RMB and one (0.33%) with two veins: one passing posterior to the RMB and another RIB on the pulmonary aspect. The frequency of right superior PV variants reported by Akiba et al. was 0.3–9.3% [3].

A review by Sato et al. in 2022 reported maximum and minimum vessel diameters of 7.0 and 2.2 ± 0.72 mm, respectively, with the RTPV usually posterior to the RIB [4]. To our best knowledge, the anomalous vessel in the present case is the largest reported RTPV to date, with a diameter of 7.8 mm. It is also an extremely rare variant, passing posterior to the RMB and draining into the LA.

Generally, subcarinal LND is mandatory in radical esophagectomy for esophageal cancer. When performing subcarinal LND, injuring the RTPV may result in massive hemorrhage [5] or cardiac tamponade due to pericardial hemorrhage [6]. However, interrupting drainage of the culprit vessel may result in segmental pulmonary congestion. Some reports have indicated that no signs of upper lobe congestion have occurred after ligation of a RTPV with a diameter ≤ 4.5 mm [7]. Adequate caution should be exercised to prevent injury to a RTPV with a diameter ≥ 4.5 mm, and ligation should be avoided.

Many reports in recent years have documented the utility of prone thoracoscopic esophagectomy in cases of cancer, and one benefit is the favorable intraoperative visibility in the middle and lower mediastinum [8]. In this case, we first mobilised the subcarinal LN from the parietal pericardium and carefully observed the RTPV while performing fractional resection of the bilateral subcarinal LN, thus facilitating safe dissection.

Preoperative 3D CT has been reported to be useful for detecting anomalous vessels in preoperative esophageal cancer cases [8, 9]. Safer surgery is achieved by esophageal surgeons keeping anomalous vessels in mind when examining the preoperative imaging and during the surgical approach.

To date, six cases of RTPV have been reported in patients with esophageal cancer. The approach was via thoracotomy [5], left lateral thoracoscopy [6] and laparoscopic transhiatal surgery [10] in one patient each. The prone thoracotomy approach was first reported by Onodera et al. in 2019 [9], and not including the present case, three cases have been reported to date [4, 8, 9]. Our case is the fourth to be reported, and these four cases are summarised in Table 1. The anomalous PV was observed on preoperative CT in all cases, facilitating subcarinal LND with vessel preservation. Thoracoscopic esophagectomy may involve areas posterior to the right upper lobe, and a prone position has been reported to be a more favorable means of securing the surgical field to confirm the presence of an RTPV [9]. We secured a favorable surgical field in the present case and safely performed subcarinal LND while preserving the RTPV.

**Table 1:** Cases of prone thoracoscopic esophagectomy for esophageal cancer in patients with a right top pulmonary vein

Author	Year	Age	Sex	Location	Origin	Inflow site	Preoperative recognition	Reconstruction route	Operative time (min)	Blood loss (ml)	Postoperative hospital stay (days)
Onodera [9]	2019	61	M	Intermediate	S2	RSPV	Yes	PMR	815	52	17
Matsubara [5]	2020	77	M	Intermediate	S2	RSPV	Yes	PMR	620	150	−
Sato [4]	2022	70	M	Main	S2	LA	Yes	RR	360	30	21
Our case	2023	60	M	Main	S2	LA	Yes	RR	394	45	17

PMR: Posterior mediastinum route

RR: Retrosternal route
